# 

**Published:** 1903-12-05

**Authors:** 


### Publications.

Just published, thoroughly Revised, with 4 Plates and 166 Illustrations, Crown 8vo., 10s. 6d.

**DISEASES OF WOMEN.** A Practical Text-book.

By Arthur H. N. Lewers, M.D.Lond., F.R.C.P.Lond., Senior Obstetric Physician to the London Hospital, and Lecturer on Midwifery in the London Hospital Medical School; Examiner in Obstetric Medicine to the University of London; Examiner in Midwifery and Diseases of Women at the Conjoint Board of the Royal College of Physicians of London and of the Royal College of Surgeons of England, &c.

[*Lewis's Practical Series.*

By the same Author, with 3 Coloured Plates and 51 Original Illustrations, 8vo., 10s. 6d. net.

**CANCER OF THE UTERUS: A CLINICAL MONOGRAPH ON ITS DIAGNOSIS AND TREATMENT.** With the AFTER-RESULTS in SEVENTY-THREE CASES treated by RADICAL OPERATION.

"The book is a treatise, pathologically, surgically, and clinically, of standard value, and deserves to be widely known and studied.—*British Medical Journal.*

London: H. K. Lewis, 136 Gower Street, W.C.

---

JUST ISSUED. EIGHTH EDITION. Crown 8vo, 10s. 6d.

**PHARMACY, MATERIA MEDICA AND THERAPEUTICS**

Is now rewritten and printed in large type, and is made a companion to the following volume. It contains an account of the action **of every drug in the B.P.**, as well as a section upon all the newest drugs, with an Introduction to the Art of Dispensing. With woodcuts, prescriptions, &c.

Also FOURTH EDITION. 1055 pages, 8vo. 16s.

**DICTIONARY OF TREATMENT.**

Containing a Revised and up-to-date Article upon every Diseased Condition, whether Medical, Surgical, Gynæcological, or Ophthalmological, as well as Articles upon Poisoning, Drowning, and the various Surgical Emergencies and Accidents, arranged for Rapid Reference.

By W. WHITLA, M.A., M.D., Professor of Materia Medica, Queen's College, Belfast; Senior Physician to the Royal Victoria Hospital, &c.

The Publisher will supply both volumes, carriage paid, for 21/-

London: HENRY RENSHAW, 356 Strand.

---

**CLINICAL LECTURES ON NEURASTHENIA.**

By THOMAS D. SAVILL, M.D.Lond. Second Edition.

"Deals very exhaustively with the subject, and will be read with great interest."—*The Lancet.*

"It should be in the hands of every practitioner who wishes to keep himself well informed on this important subject."—*Treatment.*

H. J. GLAISHER, 57 Wigmore Street, W. 5/- net; 5/4 by post.

---

Crown 8vo., price 3/6.

With a "Plea for the State Registration of Nurses."

**MOCK NURSES OF THE LATEST FASHION.**

By FREDERICK J. GANT, F.R.C.S., Consulting Surgeon to the Royal Free Hospital.

BAILLIÈRE, TINDALL & COX, Henrietta Street, Covent Garden, London.

---

WORKS BY Mr. McADAM ECCLES, M.S., F.R.C.S., Assistant Surgeon St. Bartholomew's Hospital, &c.

**HERNIA.**

Second Edition, 1902. Price 7/6 net. Illustrated by Numerous Photographs.

**THE IMPERFECTLY DESCENDED TESTIS.**

Just published. Profusely Illustrated. Price 7/6.

London: BAILLIÈRE, TINDALL & COX.

---

2nd Edition. Revised and Enlarged. Now Ready.

Post 8vo. pp. xv.-268, and 39 Illustrations in the text. Cash price 4/6, postage 4d.

**PRACTICAL TEXT-BOOK OF MIDWIFERY FOR NURSES.**

By ROBERT JARDINE, M.D. Edin., M.R.C.S. Eng., F.F.P. and S. Glas.

Professor of Midwifery in St. Mungo's College, Glasgow; Physician to the Glasgow Maternity Hospital; late President of the Glasgow Obstetrical and Gynæcological Society.

"It is the best suited for its purpose of any book of the kind which we have come across. . . . For nurses the little book is an improvement on any similar book of the kind with which we are familiar."
*The Dublin Journal of Medical Science.*

W. F. CLAY, 18 Teviot Place, Edinburgh.

---

Crown 8vo. cloth, price 5s. net; by post, 5s. 4d.

*Second Edition of*

DR. HEYWOOD SMITH'S

**PRACTICAL GYNÆCOLOGY.**

This is a genuine handbook on the subject, intended for ready reference by busy practitioners.

W. J. GLAISHER, 57 Wigmore Street, Cavendish Square, W.

---

JUST OUT. With 3 coloured Plates, and 54 other Illustrations. Price 6/- net.

**HANDBOOK OF DISEASES OF THE EAR**

For Students and Practitioners.

By RICHARD LAKE, F.R.C.S. Eng., Surgeon to the Royal Ear Hospital.

London: BAILLIÈRE, TINDALL & COX, 8 Henrietta Street, Covent Garden.

### Publications—*continued.*

**THE HANDBOOK OF THE OPEN-AIR TREATMENT**

By Dr. Charles Reinhardt

(Visiting Physician to Hailey Sanatorium, Wallingford, and Stourfield Park Sanatorium, Bournemouth),

Contains Views, Descriptions, and Information respecting 30 Sanatoria in Great Britain.

Price: Cloth, 2/6; Paper, 1/- (postage 4d.)

The "HEALTH RESORT" Publishing Office, 379 Strand, London, W.C.

Crown 8vo. Illustrated. Price **2s. 6d.** net.

**ANÆSTHETICS.** By J. Blumfeld,

M.D.Cantab., Senior Anæsthetist to St. George's Hospital; Anæsthetist to the Grosvenor Hospital for Women and Children, London, and to the National Dental and Metropolitan Hospitals.

*Lancet.*—"....In writing this handbook the author has been quite successful." *Australasian Medical Gazette.*—"We can strongly commend this book." *Medical Chronicle.*—"The book is one which we can heartily recommend to senior students and practitioners."

London: Baillière, Tindall & Cox, 8 Henrietta St., Covent Garden.

### NOTICE TO CORRESPONDENTS.

All MSS., Letters, Books for Review, and other matters intended for the Editor should be addressed The Editor, "The Hospital," The Hospital Buildings, 28 & 29 Southampton Street, Strand, W.C.

The Editor cannot undertake to return rejected MS., even when accompanied by stamped directed envelope.

**Notice to Advertisers and Subscribers.**

All Advertisements, Orders for Copies of The Hospital, and Business Communications should be addressed to **THE MANAGER (Not to the Editor),** The Hospital, 28 & 29 Southampton Street, Strand, London, W.C.

The Cost of Subscription, post free, is as follows:—Per week, 3½d.; per quarter, 3s. 9d.; per half-year, 7s. 6d.; per annum, 15s. Foreign and Colonial:—Per annum, 19s. 6d., payable, in all cases, in advance.

**NOTICE.—Vol. XXXIV. of "The Hospital" (April 1903 to September 1903), in handsome cloth binding, is now on sale at the office of this Journal, price 7s. 6d. Cases for binding the Numbers contained in this Volume 1s. 6d. Index to Vol. XXXIV., 2½d., post free.**

**The Monthly part for December is now ready. Price 1s., by post, 1s. 3d.**

### SCRAPS AND GLEANINGS.

HIS MAJESTY THE KING has been graciously pleased to send a present of two boxes of cast linen to the Cancer Hospital, Free, Fulham Road, London, S.W.

THE death is recorded of a woman in her 105th year, who up to a short time ago was in full possession of all her faculties (with the exception of being slightly deaf), and could read and knit without using spectacles.

AN excellent smoking concert was given at the Medical School, Leeds, on November 17. An admirably-drawn programme, by Mr. F. Reynolds, of somewhat gruesome design, bristling with anatomy, physiology, and medical science, formed a connecting link between the environments of the concert and the more frivolous nature of the entertainment.

### THE STUDENT OF MEDICINE.

### APPOINTMENTS.

**J. Crowley**, L.R.C.P.&S.Edin., L.F.P.S.Glasg., Certifying Factory Surgeon for the Ballycastle District, Mayo. **David Gault**, M.D., F.R.C.S., Medical Superintendent to the Orokonui Home for Inebriates, Waitati, Otago, New Zealand. **J. Donald Pollock**, M.D., C.M.Edin., Radiographer and Superintendent of the Electrical Department at the Cancer Hospital, Fulham Road, S.W. **F. Lionel Provis**, M.R.C.P., F.R.C.S.Edin., M.R.C.S.Eng., Physician to Out-patients at the Chelsea Hospital for Women. **F. U. Purchas**, M.D., C.M.Edin., Certifying Factory Surgeon for the Newtown District, Montgomeryshire. **R. R. Roger**, M.D.Canada, Clinical Assistant, Chelsea Hospital for Women. **J. Allan C. Roy**, M.B., Ch.B.Vict., Junior House Surgeon to the Manchester Royal Eye Hospital. **H. H. Rubra**, M.D.Brux, M.R.C.S.Eng., L.R.C.P.Lond., Postal Medical Officer to the Hornsey Division. **A. G. Young**, M.D., Certifying Factory Surgeon for the Castle Bytham District, Lincolnshire.

ST. THOMAS'S HOSPITAL.—The following gentlemen have been selected as House Officers from Tuesday, December 1st, 1903:—House Physicians (*Extension*): **P. N. Panton**, B.A.Cantab., L.R.C.P., M.R.C.S., and **J. N. Sergeant**, L.R.C.P., M.R.C.S. **R. E. H. Leach**, M.A., M.B., B.Ch.Oxon., L.R.C.P., M.R.C.S.; **G. R. Rickett**, M.A., B.C.Cantab. House Physicians to Out-patients: **G. C. Adeney**, L.R.C.P., M.R.C.S.; **E. A. Ross**, M.B., B.C.Cantab. House Surgeons (*Extension*): **J. E. Adams**, L.R.C.P., M.R.C.S.; **H. Upcott**, L.R.C.P., M.R.C.S.; **C. Wheen**, B.A.Oxon., L.R.C.P., M.R.C.S.; and **N. Carpmael**, L.R.C.P., M.R.C.S. House Surgeons to Out-patients (*Extension*): **W. L. Harnett**, B.A.Cantab., L.R.C.P., M.R.C.S.; **H. I. Pinches**, B.A., M.B., B.C.Cantab., L.R.C.P., M.R.C.S.; **T. Guthrie**, B.A., B.C.Cantab., L.R.C.P., M.R.C.S.; and **L. E. Wigram**, M.A., M.B., B.C.Cantab. Obstetric House Physicians: (*Senior*) **W. H. Harwood-Yarred**, B.Sc.Lond., L.R.C.P., M.R.C.S.; (*Junior*) **F. E. Shipway**, M.A., M.B., B.C.Cantab. Clinical Assistants in the Special Departments: (Throat) **A. C. Birt**, L.R.C.P., M.R.C.S.; (Skin) **C. N. Sears**, M.B.Lond., L.R.C.P., M.R.C.S. (*Extension*); **B. Higham**, L.R.C.P., M.R.C.S.; (Ear) **G. T. Birks**, B.A., B.C.Cantab. (*Extension*); **J. C. F. D. Vaughan**, L.R.C.P., M.R.C.S.

### "The Hospital" Institutional Library.

"Hospitals and Asylums of the World." 4 Vols., with a Portfolio of Plans, complete £12 12s.
"Burdett's Hospitals and Charities, 1903." 5s. net.
"Burdett's Official Nursing Directory, 1903." 3s. net.
"Cottage Hospitals, General, Fever, and Convalescent." 10s. 6d.
"Uniform System of Accounts for Hospitals and Public Institutions." New Edition. 4s. net.
"Hospital Expenditure: The Commissariat." 2s. 6d.
"A Legal Handbook for the Use of Hospital Authorities." 2s. 6d.
"The Cottage Hospital Case Book or Register of Patients." 10s. and 12s. 6d.

All these are published by the SCIENTIFIC PRESS, Ltd., and may be obtained through any bookseller, or direct from the publishers, 28 and 29 Southampton Street, Strand, London, W.C.

### Tenders.

**CANCER HOSPITAL (FREE), Fulham Road, S.W.**

THE NURSING PROFESSION: How and Where to Train.
By Sir HENRY BURDETT, K.C.B.
A reliable and up-to-date Guide to Training for the calling of a Nurse. In its pages would-be Nurses will find all the information they require.
New Edition. Price 2/-, post free 2/4.
Of all Booksellers or of THE SCIENTIFIC PRESS, LTD., 28 & 29 Southampton Street, Strand, London, W.C.

Just Published, 188pp., Crown 8vo., Price 2s. 6d.

### GYNÆCOLOGICAL NURSING.

By NETTA STEWART

Ward Sister, Edinburgh Royal Infirmary.

**With Preface by SIR HALLIDAY CROOM.**

Sir Halliday Croom says in Preface:—"It seems to me to fill an obvious want, and brings credit to the Nursing School to which the Authoress belongs. In many respects it is unique, and it is throughout useful and practical."

The *Lancet* says:—"This little book contains an excellent account of all that a gynæcological nurse ought to know. . . . The Author has been fortunate in her efforts to give sufficient prominence to those facts which are of special importance and yet not leave out anything of value."

The *British Journal of Nursing* says:—"The value of the book is increased by the excellent illustrations, which add considerably to the clearness of the text. We hope it will be added to every nurse's library, and the price brings it within the reach of most nurses to possess."

Edinburgh:—OLIVER & BOYD. London:—SIMPKIN, MARSHALL, HAMILTON & CO., LTD.

### Handbooks on District Nursing.

***JUST PUBLISHED.***

| | Price post free. |
|---|---|
| **HINTS ON HOW TO START A DISTRICT NURSING ASSOCIATION.** By A COUNTY SUPERINTENDENT. This work, which is the outcome of great personal experience, gives full particulars on how to start a District Nursing Association, and will be found of the greatest value to those who desire to establish such an institution in their own immediate neighbourhood. The information it contains will also prove exceedingly useful to all in any way interested in District Nursing. | 1/1 |
| **PRACTICAL HINTS ON DISTRICT NURSING.** By AMY HUGHES. Late of the Central Training Home, Queen Victoria's Jubilee Institute for Nurses for the Sick Poor. | 1/- |
| **DISTRICT NURSING ON A PROVIDENT BASIS.** By JAMISON B. HENRY, M.D. | 2/- |
| **THE ADVANTAGES AND PRIVILEGES OF A TRAINED DISTRICT NURSE AND THE DUTIES OF THE DISTRICT TOWARDS HER.** By SIR HENRY BURDETT, K.C.B. | 2d. |

*Of all Booksellers, or of*

THE SCIENTIFIC PRESS, LTD., 28 & 29 Southampton Street, Strand, London, W.C.

*COMPLETE CATALOGUE POST FREE ON APPLICATION.*

### J. & A. CHURCHILL'S
*Books for Nurses.*

### Suitable Presents

### For CHRISTMAS and the NEW YEAR

### NURSING.
Nursing, General, Medical and Surgical, with an Appendix on Sick Room Cookery. By WILFRED J HADLEY, M.D., F.R.C.P., F.R.C.S.. Physician and Pathologist to the London Hospital, Lecturer to Nurses at the London Hospital Nursing School . . . . . 3s. 6d.

### MEDICINE.
Lectures on Medicine to Nurses. By HERBERT E. CUFF, M.D., F.R.C.S. Medical Superintendent, North Eastern Fever Hospital London. Fourth Edition. With 29 Illustrations . . . . . 3s. 6d.

### INFANT FEEDING.
The Natural and Artificial Methods of Feeding Infants and Young Children By EDMUND CAUTLEY M.D. F.R.C.P., Physician to the Belgrave Hospital for Children; late Casualty Physician and Assistant Demonstrator of Physiology, St. Bartholomew's Hospital. Second Edition 7s. 6d.

### DIET.

Diet and Food considered in relation to Strength and Power of Endurance, Training and Athletics. By A. HAIG, M.D, F.R.C.P. Fourth Edition. 2s.

### MIDWIFERY.
A Short Practice of Midwifery for Nurses. By HENRY JELLETT, M.D. (Dub. Univ.), F.R.C.P.I., Ex-Assistant Master, Rotunda Hospital, Dublin, Extra Examiner in Midwifery and Gynæcology, Royal College of Physicians of Ireland, Extern Examiner in Midwifery, Royal University of Ireland. With 67 Illustrations . . . . . 6s.

### MONTHLY NURSING.
A Short Manual for Monthly Nurses. By C. J. CULLINGWORTH, M.D., F.R.C.P., Obstetric Physician to St. Thomas's Hospital. Revised with the assistance of M. A. ATKINSON, Matron of the General Lying-in Hospital, London. Fifth Edition. 1s. 6d.

### HEALTH OF A WIFE.
Chavasse's Advice to a Wife on the Management of her Own Health. Fourteenth Edition. By FANCOURT BARNES, M.D., Consulting Physician to the British Lying-in Hospital . . . . 2s. 6d.

### HEALTH OF CHILDREN.
Chavasse's Advice to a Mother on the Management of her Children. Fifteenth Edition. By GEORGE CARPENTER, M.D., Physician to the Evelina Hospital for Children . . . . . . 2s. 6d.

### ANTISEPTICS.

Lessons in Disinfection and Sterilisation; an Elementary Course of Bacteriology, with a Scheme of Practical Experiments By F. W. ANDREWES, M.D., F.R.C.P., Lecturer on Pathology at St. Bartholomew's Hospital. With Illustrations . 3s. net. [*Just Published.*

### SURGERY.
A Manual of Minor Surgery and Bandaging. By CHRISTOPHER HEATH, F.R.C.S., and BILTON POLLARD, F.R.C.S., Surgeon to University College Hospital. Twelfth Edition. With 195 Illustrations. 6s. 6d.

### HYGIENE.
The Elements of Health: An Introduction to the Study of Hygiene. By LOUIS C. PARKES, M.D., D.P.H.Lond., Lecturer on Public Health at St. George's Hospital. 3s. 6d.

### DICTIONARY.
A Short Dictionary of Medical Terms. By R G. MAYNE M.D., LL.D. . 2s. 6d.

7 GREAT MARLBOROUGH STREET, LONDON

